# Medical Management after Lancehead Snakebite in North Amazon: A Case Report of Long-Term Disability

**DOI:** 10.3390/toxins14070494

**Published:** 2022-07-16

**Authors:** Isadora S. Oliveira, Carla B. Ananias, Jilvando M. Medeiros, Michelle V. S. Franco, Isabela G. Ferreira, Felipe A. Cerni, Eliseu A. Sandri, Wuelton M. Monteiro, Manuela B. Pucca

**Affiliations:** 1 Department of BioMolecular Sciences, School of Pharmaceutical Sciences of Ribeirão Preto, University of São Paulo, Ribeirão Preto 14040-903, Brazil; isadora_so@yahoo.com (I.S.O.); igobboferreira@usp.br (I.G.F.); 2 Medical School, Federal University of Roraima, Boa Vista 69310-000, Brazil; carlabalenzuela@gmail.com (C.B.A.); jilvandom@gmail.com (J.M.M.); michelle.franco56@gmail.com (M.V.S.F.); 3 Health Sciences Posgraduate Program, Federal University of Roraima, Boa Vista 69310-000, Brazil; felipe_cerni@hotmail.com; 4 Insikiram Institute of Indigenous Higher Studies, Federal University of Roraima, Boa Vista 69310-000, Brazil; eliseu.sandri@ufrr.br; 5 Department of Teaching and Research, Dr. Heitor Vieira Dourado Tropical Medicine Foundation, Manaus 69040-000, Brazil; wueltonmm@gmail.com; 6 Department of Medicine and Nursing, School of Health Sciences, Amazonas State University, Manaus 69050-010, Brazil

**Keywords:** snakebite envenoming, *Bothrops*, Roraima, necrosis, sequelae

## Abstract

Snakebites are a major public health problem in indigenous communities in Brazil, leading to acute local and systemic damage with resulting deficiencies. Long-term musculoskeletal disabilities related to snakebites have been a neglected area of research. *Bothrops* (lancehead) snakes are responsible for most of the permanent sequelae related to snakebites in Latin America. Here, we present a case report of a 32-year-old male indigenous patient who was envenomed by a *Bothrops* species. The patient was clinically followed for a period of approximately 2 years and 6 months, during which time he experienced a loss of musculoskeletal tissue and required several medical procedures such as debridement, tissue reconstruction, and physical therapy, which resulted in a recovery of mobility, though with a permanent sequelae in gait. This case report shows how snakebites have a significant impact on health systems, as victims require physiotherapy, plastic surgery, and orthopedics services, as well as social support for reintegration into their local communities.

## 1. Introduction

Snakebite envenomings kill more than 100,000 victims each year and maim more than 400,000 others [1]. As a result, in 2017, the World Health Organization (WHO) classified snakebites into Category A of the Neglected Tropical Diseases [2]. Snakebites mainly affect the poor, rural workers, and indigenous people from developing countries in Africa, Latin America, and Asia [3,4,5].

In Brazil, snakebites are also considered an important public health problem. According to the Brazilian *Sistema de Informações de Agravos de Notificação* (SINAN) reports, the country sees approximately 30,000 snakebites per year. Most snakebites (~70%) in Brazil are caused by members of the *Bothrops* genus, and, fortunately, most of these envenomings are resolved (Table 1) [6]. There are 29 species of *Bothrops* (lancehead) snakes distributed throughout the Brazilian territory [7], with the northern region being the most impacted by these accidents. Indeed, the north of Brazil is inhabited by several species, such as *Bothropsatrox*, *B. bilineatus*, *B. brazili*, *B. lutzi*, *B. marajoensis*, *B. marmoratus*, *B. mattogrossensis*, *B. moojeni*, *B. oligobalius*, and *B. taeniatus* [7].

The venom of *Bothrops* spp. can trigger proteolytic, hypotensive, and coagulant actions (i.e., blood incoagulability). In addition, it presents hemorrhagins, which damage capillaries and cause hemorrhages, and, in combination with coagulation disorder and thrombocytopenia, it can aggravate hemorrhagic syndrome [8,9,10]. Locally, *Bothrops* snakebite is characterized by edema (which appears quickly after the bite), pain, and myonecrosis, as well as intense local inflammation [11,12,13,14].

The victim can present complications such as acute kidney injury (AKI), hepatic hematoma, and shock [15,16,17]. According to the set of signs and symptoms, snakebite severity can be classified as either mild, moderate, or severe, which directly influences the therapeutic approach, especially in regard to selecting the best antivenoms and, consequently, the number of ampoules [18].

Local complications include infection, abscesses, cellulitis, and erysipelas, mostly in moderate and severe cases. Isolated bacteria in abscesses include Gram-negative bacilli, mainly *Morganella morganii* [19]. Compartment syndrome, though rare, is severe and difficult to manage. It results from the compression of the vascular-nervous bundle resulting from severe swelling in the affected limb, producing ischemia, which may lead to necrosis. Necrosis can also be caused by infection, thrombosis, application of tourniquets, and extremity (finger) involvement, increasing the chance of functional loss or even amputation of the affected limb [18].

In addition, systemic complications include bleeding in pre-existing skin lesions; hemorrhages, such as epistaxis, gingivorrhagia, hematemesis, and melena; sepsis; and disseminated intravascular coagulation (DIC). Death may occur due to AKY, severe bleeding, sepsis, DIC, or shock [19].

Thus, we describe herein a case report with the critical clinical manifestations following *Bothrops* sp. snakebite in Roraima state (Brazil), followed by severe complications to the victim’s life.

## 2. Case Report

**Figure 1:**
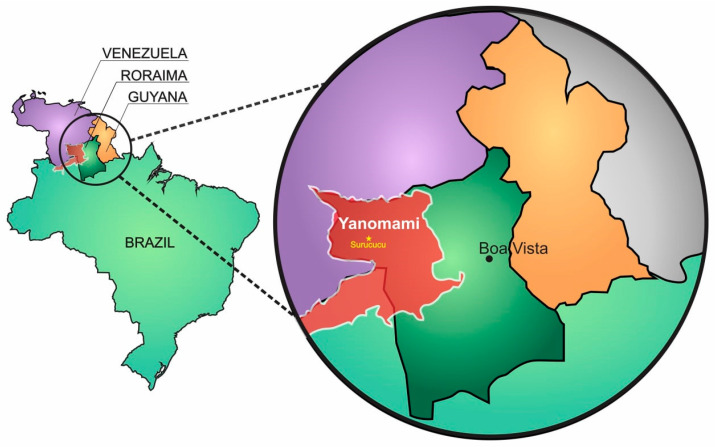
Yanomami Indigenous Land in northern Brazil. The Yanomami Indigenous Land (in red) occupies an area of 9665 hectares (96.65 km^2^) inhabited by the Yanomami and Ye’kwana indigenous communities. The Yanomami community population stands at 26,780 people (2021) and is the largest relatively isolated tribe in South America. The victim of the case report inhabits the region of Surucucu, which is where snakebite occurred (yellow star).

A 32-year-old indigenous male, living in Roraima National Forest inside demarcated Yanomami indigenous territory (near Surucucu, Roraima state, Brazil) (Figure 1), was bitten by a *Bothrops* sp. snake (locally known as “jararaca”) on the lateral side of his right leg. The accident occurred when the man was hunting in the Amazon Forest with his son, at approximately 19:30 on 8 July 2018 (day 0), and the victim declared that no symptoms were noticed initially.

Thirteen days after the accident, the victim sought medical attention at the indigenous health care unit, referring to severe pain, nausea, persistent vomiting, right leg edema, an extensive necrosis area, hyperthermia, and amber-colored urine. Immediately, the patient received intravenous hydrocortisone and intramuscular promethazine (to avoid possible hypersensitivity reactions caused by heterologous antivenoms) following intravenous (i.v.) administration of twelve ampoules of anti-bothropic antivenom. In addition, antibiotic therapy was initiated with intravenous ceftriaxone and oral metronidazole. After that, the patient was referred to the *Hospital Geral de Roraima* (HGR), the main hospital in the city of Boa Vista (the state capital).

**Figure 2:**
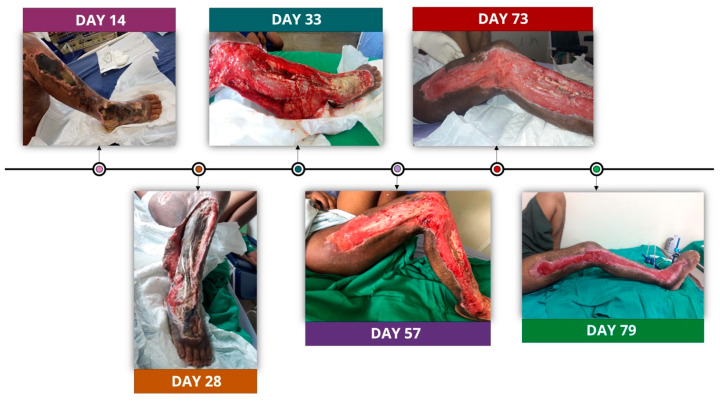
Timeline of snakebite injury of lower right limb. Necrosis evolution (day 14), lateral fasciotomy (days 28 and 33), wound reconstruction (days 57 and 73), and presence of an external fixator.

On day 14 (22 July 2018), the patient arrived at HGR complaining of intense pain. At the physical examination, the patient was presenting in regular condition (i.e., eupneic, hypoxic, anicteric, acyanotic, afebrile, and hydrated), with arterial pressure of 90/50 mmHg, a heart rate of 96 bpm, a respiratory rate of 22 irpm, and a temperature of 36.8 °C. At that moment, hospital staff discovered significant edema and an extensive necrosis area in the lower right limb: the lateral side of the thigh, calf, and dorsum of the foot (Figure 2). Analgesia was attained with tramadol, and antibiotic therapy was continued. Beyond that, due to the leg’s wound complexity, an evaluation from different specialists was requested to verify the possibility of compartment syndrome and potential need for amputation. A vascular specialist identified peripheral perfusion of the limb and discouraged amputation. However, the vascular clinician requested an evaluation from a team of surgeons.

**Figure 3:**
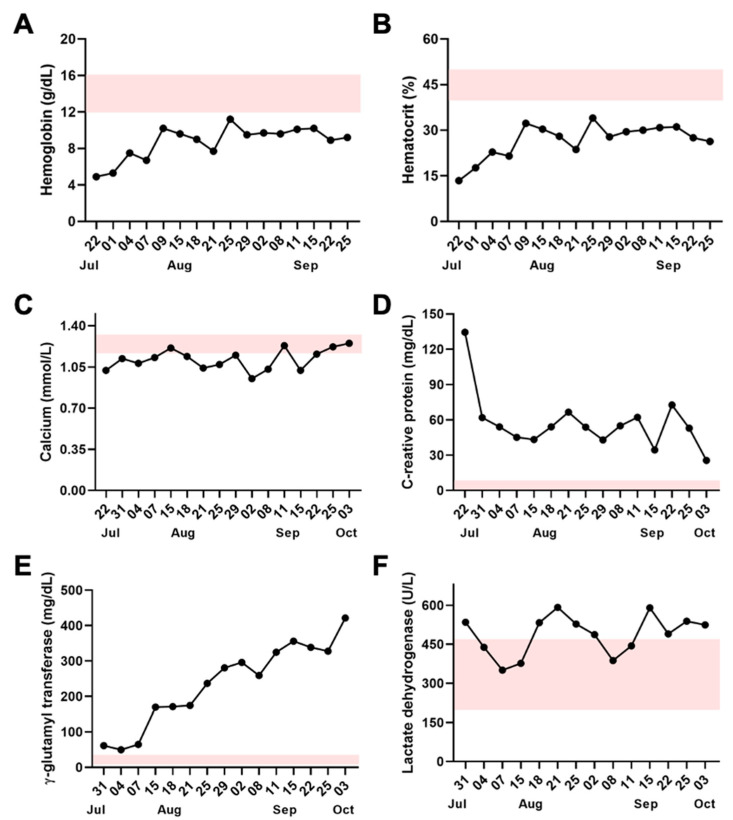
Laboratory analysis of the patient’s blood over months. Serum levels of (**A**) hemoglobin, (**B**) hematocrit, (**C**) calcium, (**D**) C-reative protein, (**E**) γ-glutamyl transferase, and (**F**) lactate dehydrogenase. Red spaces show normal values, which highlight that the patient’s serum levels are lower (**A**,**B**), and higher (**D**,**E**) than the reference range or vary between normal and altered values (**C**,**F**). Pink shade represents the reference range according to *Laboratório Central de Roraima* (LACEM—HGR), Boa Vista, Roraima, Brazil: hemoglobin: 12–16 g/dL; hematocrit: 40–50%; calcium: 1.17–1.32 mmol/L; C-reative protein: 0.0–8.0 mg/dL; γ-glutamyl transferase: 12–45 mg/dL; lactate dehydrogenase: 200–480 U/L.

The laboratorial exams showed that some parameters were normal (e.g., creatinine, platelets, alanine aminotransferase, aspartate aminotransferase, direct/indirect and total bilirubin, glucose, prothrombin time, and partial thromboplastin time); however, other parameters were outside of the reference range (Figure 3).

On day 17 (25 July 2018), the patient was evaluated by a surgeon, who mentioned the need for surgical management. However, no initiative was taken. On day 19 (27 July 2018), the patient was complaining of severe pain, particularly in his leg, with notable edema, an extensive area of necrosis, and foul-smelling secretion. An occlusive curative with collagenase ointment (for better delineation of the necrosis area) was initiated, with further reevaluation after 48 h. On day 23 (31 July 2018), the surgery team reassessed the patient and new pre-operative laboratory tests were requested, since the surgeon considered whether to perform debridement of the area.

On day 25 (2 August 2018), due to patient anemia, two blood bags were administered to the patient, and the debridement procedure was scheduled for 5 August 2018. On the following day, the patient was transferred to the infectiology department. At physical examination, extensive necrosis was observed in the right lower limb, and the wound had fetid secretion, myiasis (parasitic infestation) on the dorsum of the foot, and calcaneus with liquefactive necrosis. On day 28 (5 August 2018), the patient was referred to a surgical center. An incision on the right thigh necrosis (from right leg extending to right foot) was performed, and the devitalized tissue was removed from the surface. Extensive areas of deep necrosis and soft tissue were identified. In addition, several washes with physiological saline and chlorhexidine degermant were performed, followed by the use of a compressive curative.

Following debridement surgery, vascular surgery and orthopedics were recommended, as the extensive necrosis area resulted in the absence of distal pulses in the right lower limb. Nevertheless, the vascular specialist was able to maintain distal perfusion without compromising the vascular fibular areas and posterior tibial nerve bundles. On day 30 (7 August 2018), the patient was evaluated by an orthopedic surgeon, who identified the presence of extensive tissue damage with exposure of the tendon area in the right leg, as well as muscle injury due to severe infection with involvement of the joint area with foul-smelling necrosis. The specialist considered whether to perform limb amputation.

Based on the clinical situation of the patient, an infectious disease specialist replaced antibiotic therapy (previously ceftriaxone and metronidazole) with meropenem and teicoplanin, maintaining analgesia with opioids and dipyrone (1 g) every 4 h, and a new infusion of three bags of red blood cells was administered to the patient.

On day 33 (10 August 2018), after a new evaluation from the vascular surgeon, amputation was again discouraged. However, a new procedure was performed by the vascular surgery team. The procedure included the removal of soft tissues on the side of the thigh and right knee; removal of the anterior tibial bundle with gangrene; removal of devitalized tissues from the dorsum of the right foot; lavage with saline solution and chlorhexidine degermant; and hemostasis and occlusive dressing with hydrocolloid/alginate. Curative changes using physiological solutions and chlorhexidine degermant, as well as occlusion with collagenase, were performed in the surgical center under analgesia and sedation every two days. 

On day 47 (24 August 2018), the vascular surgery team performed surgical debridement of devitalized tissues of the thigh, leg, and right foot. After that, the patient presented exposure of the distal part of the fibula and ankle joints, which were washed with saline solution and chlorhexidine, and then covered with collagenase and bandages.

On day 57 (3 September 2018), the patient appeared to be clinically well, with less pain and improvement to the wound, so the presence of epithelization and the use of teicoplanin were discontinued. After 73 days following the accident (19 September 2019), the use of meropenem was also suspended; that is, all antibiotic therapy was discontinued. However, analgesia was maintained with opioids and dipyrone every 4 h.

On day 79 (25 September 2018), the patient was referred to the surgical center under analgesia and sedation by an orthopedic group, and surgery was performed to insert an external fixator on the right foot, given the loss of tendons and muscle tissue. The patient presented a satisfactory evolution of the clinical case during the hospitalization period. He was discharged from the hospital after 86 days following the snakebite (2 October 2018), with analgesia continued via 1g dipyrone orally every 6 h. However, he had to return to the institution three times per week to receive curatives. On day 92 (8 October 2018), the patient was referred to the orthopedic and plastic surgery services of an outpatient clinic so as to maintain use of curatives every two days at the state secondary service.

On day 122 (7 November 2018), the patient began following a curative treatment plan at the outpatient level. He presented an excellent evolution of tissue epithelization, maintaining the same frequency of curative change and referring to improvement in the level of pain, but analgesia continued with dipyrone.

**Figure 4:**
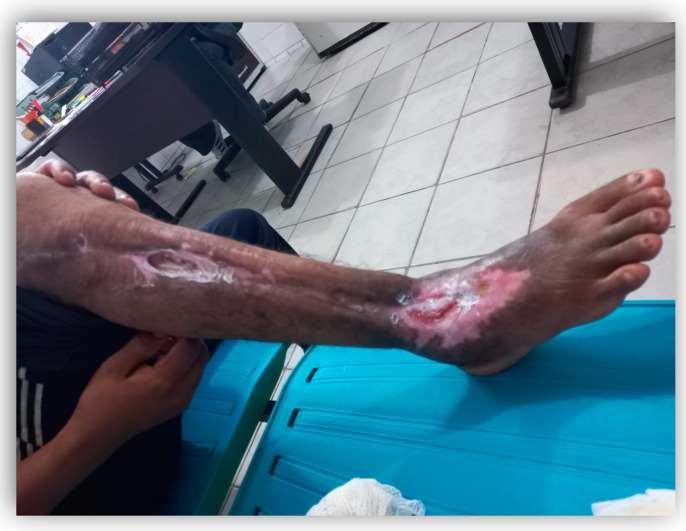
Snakebite wound indicating infection approximately 2 years after the accident (photo from 24 January 2022).

After 30 months (2.5 years) following the snakebite accident, our research group searched for the indigenous patient to see if the victim retained any snakebite sequelae. Thus, with the assistance of a nurse working on the indigenous Yanomami territory, we were able to find and evaluate the recovered patient. Surprisingly, at the evaluation, the patient presented infection at the wound (Figure 4) and was referred for treatment in Boa Vista city at the *Casa de Saúde Indígena* (CASAI, Health Home of Indigenous People—CASAI). The infection was treated and controlled, but the Yanomami patient has permanent sequelae in gait and locomotion, even after all therapeutic interventions (Video S1).

## 3. Discussion

Envenomings caused by venomous animals in Brazil have been increasing in recent years since these animals have been able to adapt to urban environments, making people more at risk of being victims of these animals [20]. In Brazil, snakebites occur in high numbers every year [6], mainly in men [3], with most cases affecting the lower limbs (e.g., legs and feet) [21], as was reported in the case presented here.

Interestingly, the victim of this study sought medical care 13 days after the bite. Given that the victim is indigenous, he may have sought the use of home medicine, such as traditional healers, before searching proper care. This delay may be inferred as negatively impacting the victim’s treatment, particularly with the delay in antivenom administration [2]. Regarding antivenom therapy, the victim received twelve ampoules of antivenom, which allowed us to classify the accident as a severe bothropic snakebite envenoming [18].

In addition, the patient arrived at the medical service with the right lower limb totally swollen and complaining of intense pain. These are classical symptoms of envenomings by *Bothrops* snakes, which become visible 2–4 h after the bite [18,22]. In addition, sloughing of the necrotic tissue was observed, and this signal usually develops weeks or even months after the bite, leading to secondary tissue infections [22].

Accidents caused by *Bothrops* snakes are common in the northern region of Brazil [23]. In Roraima, lancehead species of *B. atrox*, *B. bilineatus*, and *B. taeniatus* can be found [7]. Lancehead envenomings are usually proceeded with disseminated intravascular coagulation [18], bleeding on pre-existing skin wounds, and hemorrhages (in gums, hematuria, [24] and epistaxis), which are mainly caused by metalloproteases, phospholipases A_2_, and serine proteases [25,26,27]. These manifestations occur 6 or 8 h after the bite [11]; however, not all patients develop systemic bleeding [28]. The patient from this case report did not present bleeding signals except for hematuria, although the start time of this was not identified, as the patient arrived at the hospital 13 days after the bite.

Cases of myonecrosis are estimated to occur in 10% of *Bothrops* snakebites [11], which could explain the patient’s hematuria. The patient was medicated in the community with anti-inflammatory therapy, an approach indicated for *Bothrops* envenoming cases. A study has described that the inflammatory reaction caused by *B. moojeni* venom can be mediated by eicosanoids, histamine, and nitric oxide, and that the use of anti-inflammatory drugs can serve to reduce the edema, pain, and muscle damage caused by the venom [29].

It is important to emphasize that the victim from this case presented both permanent anatomical and functional sequelae that will last throughout his life. Although snakebite numbers are often quantified, their severity, consequences (e.g., disabilities), and impacts are still unknown [22,30,31]. Recently, using the World Health Organization Disability Assessment Schedule 2.0 (WHODAS 2.0), Aglanu et al. (2022) demonstrated that 35% of snakebite envenomings in regions of Ghana resulted in mild/moderate disabilities [32].

In addition, indigenous victims of snakebites in Brazil that developed sequelae, such as cognition level and mobility, may also develop psychosocial problems due to their frequent withdraw from the community due to feelings of shame and incapacity [33].

## 4. Conclusions

Following a snakebite, it is extremely important that the victim immediately seeks medical care in order to reduce the risk of severe sequelae, such as amputation, motility disability, and even death. Long-term snakebite disabilities need to be considered a public health problem since they have a great impact on both the victim’s life and society.

## Figures and Tables

**Table 1:** Snakebites in Brazil caused by *Bothrops* snakes.

Year	Snakebites	*Bothrops* spp.	Clinically Resolved (%)
2012	28.339	20.448	86.7
2013	27.291	19.816	87.2
2014	26.145	18.827	86.9
2015	27.113	19.560	85.9
2016	26.561	18.802	84.9
2017	28.754	20.299	85.8
2018 ^1^	29.031	20.132	86.5
2019 ^1^	32.276	22.215	86.1
2020 ^1^	31.149	21.668	83.4
2021 ^1^	29.152	20.487	81.5

^1^ Data subject to review [6].

## Data Availability

Not applicable.

## Supplementary Materials

The following supporting information can be downloaded at https://www.mdpi.com/article/10.3390/toxins14070494/s1, Video S1: Snakebite-induced permanent sequelae in gait and locomotion.
